# Face Masks Use and Its Role in Restraining the Spread of COVID-19 Pandemic in Saudi Arabia: Knowledge, Attitude, and Practices Based Cross-Sectional Study

**DOI:** 10.3389/fpubh.2021.818520

**Published:** 2022-01-24

**Authors:** Sultan Ayoub Meo, Sara A. Alqahtani, Ghada M. Aljedaie, Fatimah S. Binmeather, Renad A. AlRasheed, Raghad M. Albarrak

**Affiliations:** Department of Physiology, College of Medicine, King Saud University, Riyadh, Saudi Arabia

**Keywords:** face mask, SARS-CoV-2, COVID-19, knowledge, attitude, practice, Saudi Arabia

## Abstract

Face masks (FM) play a role in limiting the spread of viral infections; despite this, their role is influenced by the population's adherence to wearing the FM. However, the impact on the effectiveness of the FM is variable in various communities. This study aimed to investigate the knowledge, attitude, and practices toward FM use during COVID-19 in Saudi Arabia. This observational “cross-sectional questionnaire-based study was conducted in the Department of Physiology, College of Medicine, King Saud University, Riyadh, Saudi Arabia.” The data was collected using an online questionnaire survey from September 8–21, 2021, during the COVID-19 pandemic. The questionnaire was distributed *via* social media platforms to assess knowledge, attitude, and practices using single choice questions and a five-point Likert scale. Among 1,356, respondents' the rate was 678 (50%), 207 (30.5%) were males and 471 (69.5%) females. Among the participants, Saudi citizens were (649; 95.7%), with University education (502; 74%) and were mostly (368; 54.3%) between 16 and 24 years of age. The participants (384; 56.6%, *p* < 0.001) had good knowledge about face masks, and more than half of the respondents, 531 (78.3%) (*p* < 0.001), showed a positive attitude. Most of the respondents (477, 70.2%) believed that everyone could use the face mask to minimize the spread of the disease; however (111; 16.4%) reported that they would not wear a face mask if the government did not recommend wearing it in public places during the COVID-19 pandemic. Saudi citizens have above-average knowledge and optimistic attitudes toward using face masks during the COVID-19 pandemic. The community is convinced about the face masks and believes that face masks play a predominant role in limiting the spread of SARS-CoV-2.

## Introduction

The “Severe Acute Respiratory Syndrome Coronavirus 2 (SARS-CoV-2)” is a highly contagious, challenging, and threatening illness that swiftly spreads worldwide (1). The World health organization (WHO) declared COVID-19 a global pandemic and considered it the fifth pandemic globally since the Spanish flu pandemic in 1918 (2). The SARS-CoV-2 virus is transmitted through direct contact with an infected person (3). As of October 29, 2021, worldwide, the total number of SARS-CoV-2 cases is 245,373,039, and deaths 45,979,421 (2.02%). However, in Saudi Arabia, which represents a small percentage of the global number of confirmed cases, 548,475 (0.22%), and deaths 8,788 (0.01%) of the global cases and deaths (4).

Since the first case in KSA, the Saudi authorities have managed the COVID-19 pandemic by setting and following protocols to secure their citizens' lives. Which significantly contributes to minimizing and limiting the pandemic around KSA cities (5). Since the beginning of the outbreak, strict policies have been imposed, including lockdown, social distancing, praying at home, suspended all public transport (5). All of that played significantly in shaping the curve of COVID-19 cases and deaths in KSA. However, the authorities started restoring normalcy with time by raising the quarantine procedures, opening some economic and commercial activities, and praying in the mosque and public places, which increased the importance of other preventive methods such as a face mask. Wearing face masks has been imposed in the Kingdom of Saudi Arabia. Face masks play a predominant role in limiting the spread of the viral infection SARS-CoV-2. Face masks are an easy and simple tool to minimize the spread of disease, but the public knowledge, attitude practices, and population's adherence to face masks varied across the countries (6). This study investigates the knowledge, attitude, and practices of using face masks during the COVID-19 pandemic in Riyadh, Saudi Arabia.

## Subjects and Methods

This analytical observational “cross-sectional questionnaire-based study was conducted in the Department of Physiology, College of Medicine, King Saud University, Riyadh, Saudi Arabia.” The present study calculated the sample size to be 601, to achieve a 96% confidence level and a 4% margin of error. An additional 20% was added to the sample size to ensure a reasonable response rate, resulting in a sample of 721 adults in Riyadh, Saudi Arabia. We have selected 678 (50%) out of 1,356 responses randomly.

### Questionnaire and Data Collection

The data were collected using an online survey taken from September 8–21,2021, during the COVID-19 pandemic. A well-structured and pretested questionnaire was developed to achieve the study outcomes per the study's objectives. A research group established the questionnaire after a wide-ranging review of the current literature. The questionnaire items were modified from two previously published studies (7, 8). The questionnaire was distributed via social media platforms among the people in Riyadh, Saudi Arabia, to assess knowledge, attitude, and practices using a five-point Likert scale and single-choice questions. “The questionnaire consisted of five sections,” demographic variables, knowledge, attitude, practices, and compliance with wearing the facemasks “and other questions were allied to types and sources of face masks.”

Before conducting a pilot test, questions were validated through a senior faculty and these faculty members who were not included in the study. The findings gathered from the pilot assessment were only for the internal consistency reliability of the questionnaire. All the Saudi participants above 16 years of age who had access to the internet were invited. The questionnaire was administered to the participants by a web link through various social media applications.

Eight questions were used on the knowledge aspect, 3 were single choice questions, and 5 were five-point Likert scale. Two questions assessed the attitude, one single-choice and one five-point Likert scale. Lastly, four questions were used to evaluate the practice: three single-choice questions and one five-point Likert scale. The internal consistency of the questionnaire was measured by the test-retest reliability, where a coefficient of ≥0.8 demonstrated acceptable internal consistency. We added an Arabic questionnaire and the original English questionnaire to ensure that the participants fully understood the question.

### Ethical Approval

The “Institutional Review Board approved this study, College of Medicine Research Centre, King Saud University, Riyadh, Saudi Arabia” (Ref. E-21-6125).

### Statistical Analysis

The “data were analyzed using the Statistical Package for the Social Sciences (SPSS) version 26 (IBM Corp., Chicago, Illinois, USA).” Univariate analysis was applied after random sampling of respondents to calculate frequency (*n*) and percentage (%) for all categorical variables. For the analysis of continuous variables, mean ± standard deviation (SD) was measured.

There were eight knowledge, two attitude, and four practice-based questions in the questionnaire, and each correct answer was marked one point. The mean score of each component for knowledge, attitude, and practices (KAP) was considered a cut-off value. Respondents' score equal to or higher than the cut-off value was regarded as a good level of each of the three components of KAP, while an individual's score lower than the cut-off value indicated a poor level. To calculate the significance between demographical characteristics with KAP, the Chi-square test for trend and Pearson Chi-square test was used, and a *P* ≤ 0.05 was considered significant.

## Results

### Socio-Demographic Characteristics of Study Participants

Among all 1,356 respondents, 678 (50%) were randomly selected for the study, including 207 (30.5%) males and 471 (69.5%) females. Of all the participants, most of them were Saudi citizens (649; 95.7%) with University education (502; 74%) and were mostly (368; 54.3%) between 16 and 24 years of age. The average household income of all the subjects was between 10,001 and 20,000 SAR per month (Table 1).

**Table 1:** Demographic characteristics, level of education, employment, and income status of 678 subjects.

**Characteristics**	**Summary statistics *N* (%)**
**Age (years)**	
16–24	368 (54.3)
25–34	113 (16.7)
35–44	74 (10.9)
45–54	90 (13.3)
55–64	29 (4.3)
65–74	2 (3)
75 and Above	2 (3)
**Gender**	
Male	207 (30.5)
Female	471 (69.5)
**Education Status**	
Primary School	4 (6)
Elementary School	6 (9)
High School	105 (15.5)
University	502 (74.0)
Postgraduate	53 (7.8)
Other	8(1.2)
**Employment status**	
Student	325 (47.9)
Employed	199 (29.4)
Unemployed	79 (11.7)
Retired	50 (7.4)
Other	25 (3.7)
**Nationality**	
Saudi	649 (95.7)
Non-Saudi	29 (4.3)
**Income (SR)/month**	
Below 5,000 SAR	99 (14.6)
5,001–10,000 SAR	138 (20.4)
10,001–20,000 SAR	154 (22.7)
20,001–40,000 SAR	136 (20.1)
40,001–60,000 SAR	57 (8.4)
Greater than 60,001	94 (13.9)

### Knowledge, Attitudes, and Practices Related to Face Mask Use

**Figure 1:**
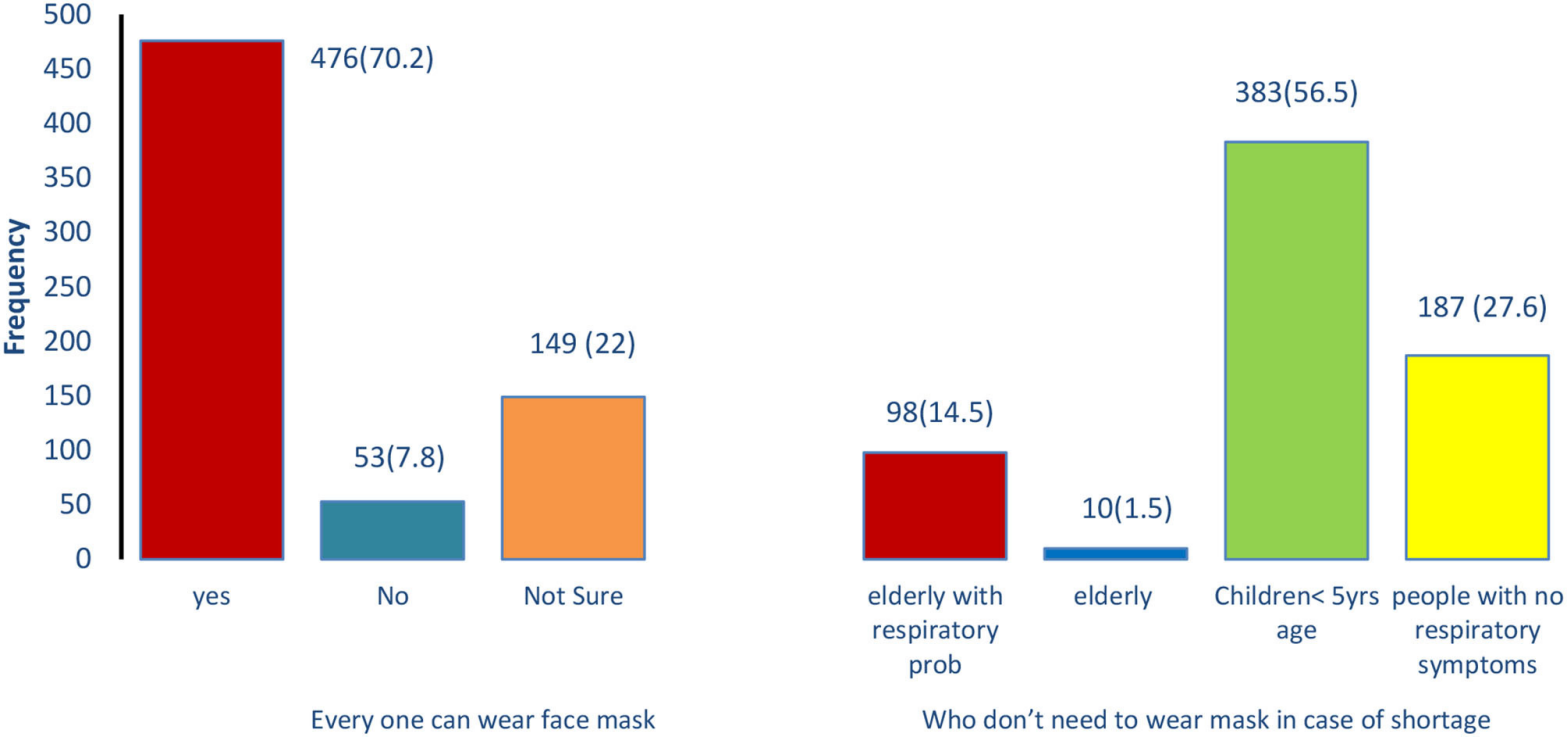
Participant's knowledge of wearing the face mask.

Among all the participants, a large number of individuals (384; 56.6%; *p* < 0.001) had a good knowledge score of face masks used with a mean score of 6.2 ± 1.2 (*p* < 0.001) (Table 2). The mean score of attitudes toward face mask use was 0.8 ± 0.47 (*p* < 0.001), and more than half of the respondents, 531 (78.3%) (*p* < 0.001), showed a positive attitude. The study observed a good practice score (460; 67.8) (*p* < 0.001) in the majority of the participants, with an average score of 2.7 ± 0.085 (*p* < 0.001). Most of the respondents (476; 70.2%) believed that everyone could use the face mask to minimize the spread of the disease. However, 149 (22%) participants were unaware of who could use it, and a significant difference was observed among males and females (*p* = 0.09). A significantly greater number (*p* = 0.02) of participants (females:282, and males:101) reported that children under 5 years of age don't need to wear masks in case of severe shortage, while fewer respondents had other concepts (Figure 1).

**Table 2:** Knowledge, attitudes, and practices of 678 respondents related to face mask use and reuse.

**Items**	**Mean ±SD or n (%)**
Knowledge score	6.2 ± 1.2 (1–8)
Knowledge score group	
Poor	294 (43.4)
Good	384 (56.6)
Attitude score	0.8 ± 0.47 (0–2)
Attitude score group	
Negative	147 (21.7)
Positive	531 (78.3)
Practice score	2.7 ± 0.085 (0–4)
Poor	218 (32.2)
Good	460 (67.8)
Why reuse face mask the mask is still clean (there is no visible dirt)	197 (29.1)
Reusing the face mask in accordance with the manufacturer's recommendation	66 (9.7)
Saving money	25 (3.7)
I do not reuse my face mask	361(53.2)
Face masks minimize the disease spread	477 (70.2)
Other	29(4.3)

*SD, standard deviation*.

**Figure 2:**
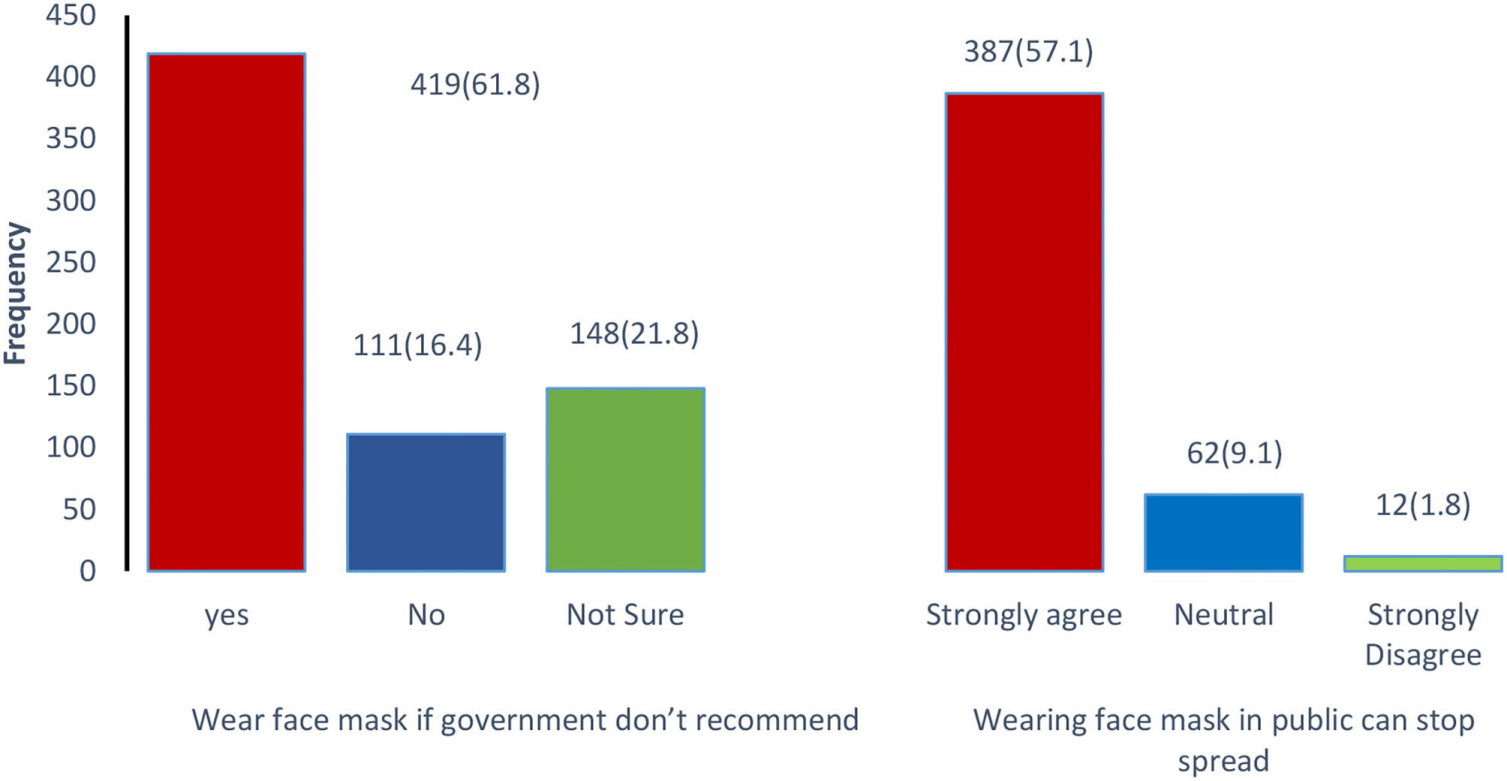
Participant's attitude toward using a face mask.

**Figure 3:**
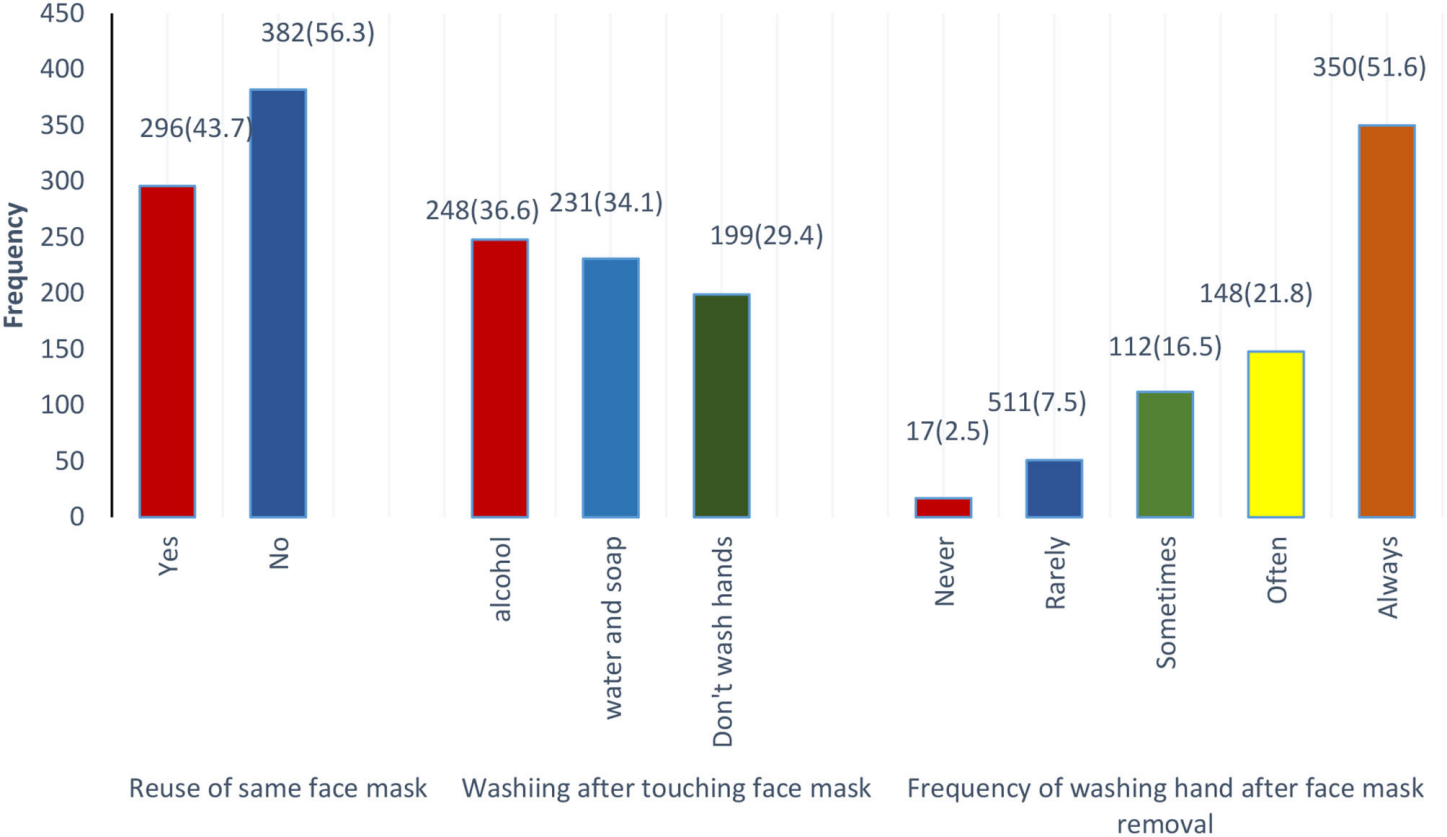
Participant's practice using a face mask.

Out of all the individuals, only a few (111; 16.4%) reported that they would not wear a face mask if the government did not recommend wearing it in public places during the COVID-19 pandemic. However, most of them showed compliance with the face mask and preferred to use it despite the government's non-recommendations (Figure 2). The majority of the males and females (350; 51.6%) follow good hygiene practices and always wash their hands after removal of face mask (*p* = 0.02) with alcohol-based hand rub (248; 36.6%; *p* = 0.3) or soap and water (*n* = 231; 34.1%; *p* = 0.06) however, only 29.4% (*p* = 0.1) of the respondents were not in the practice of washing hands after face mask removal. The study also observed that among male and females 88.8% (602; *p* = 0.70) of the respondents use surgical masks, and only 2.4 % (*n* = 16) use N95 (*p* = 0.627) (Figure 3).

Of the all males and females face mask users, 296 (43.7%) reported that they reused face masks and had a perception that the face mask is still clean if no visible dirt (196; 29.9%; *p* = 0.018), while some of them (66; 9.7%; *p* = 0.084) reuse it according to the manufacturer recommendations. Although, most of the study population (382; 56; 1%; *p* = 0.061) showed the good practice of not wearing the same mask again (Table 2).

### Factors Affecting Knowledge, Attitudes, and Practices Related to Face Mask Use

There was no significant difference in knowledge among various age groups and nationalities. However, a significant difference was observed in the different income groups of respondents (*p* = 0.003). Gender variations (*p* = 0.021) and education level (*p* = 0.04) significantly changed the attitudes toward wearing a face mask, and a positive attitude was observed primarily on University students. There was a significant difference in the practices of using a face mask in different age groups (*P* < 0.001), an education level (*p* = 0.04), and income group (*p* = 0.029), while there was no effect of gender and nationality on the practice of using face masks (Table 3).

**Table 3:** Association of knowledge, attitude, and practices of face mask use.

**Characteristics**	**Knowledge**		**Attitude**		**Practice**	
	**Good *N* (%)**	**Poor *N* (%)**	**Positive *N* (%)**	**Negative *N* (%)**	**Good *N* (%)**	**Poor *N* (%)**
Age	*P* = 0.508^a^		*P* = 0.564^a^		*p* < 0.001^a^	
16–24	204 (30.1)	164 (24.2)	288 (42.5)	80 (11.8)	229 (33.8)	139 (20.5)
25–35	65 (19.6)	48 (7.1)	85 (12.5)	28 (4.1)	82 (12.1)	31 (4.6)
35–44	45 (6.6)	29 (4.3)	62 (9.1)	12 (1.8)	52 (7.7)	22 (3.2)
45–54	46 (6.8)	44 (6.5)	67 (9.9)	23 (3.4)	66 (9.7)	24 (3.5)
>55	24 (3.5)	9 (1.3)	29 (4.3)	4 (0.6)	31 (4.6)	2 (0.3)
Gender	*P* = 0.835^b^		*p* = 0.0216^*^^b^		*p* = 0.250^b^	
Male	116 (17.1)	91 (13.4)	156 (23.0)	51 (7.5)	134 (19.8)	73 (10.8)
Female	268 (39.5)	203 (29.9)	375 (55.3)	96 (14.2)	326 (48.1)	145 (21.4)
Nationality	*p* = 0.192^b^		*p* = 0.430^b^		*p* = 0.895^b^	
Saudi	368 (54.3)	281 (41.4)	510 (75.2)	139 (20.5)	440 (64.9)	209 (30.8)
Non Saudi	6 (2.4)	13 (1.9)	21 (3.1)	8 (1.2)	20 (2.9)	9 (1.3)
Education	*p* = 0.518^a^		*p* = 0.040^aa^		*p* = 0.04^*^^a^	
Secondary and	68 (10.0)	47 (6.9)	91 (13.3)	24 (3.4)	86 (12.7)	29 (4.3)
Higher Secondary						
University	279 (41.2)	223 (32.9)	394 (58.1)	108 (15.9)	330 (48.7)	172 (25.4)
Postgraduate	37 (5.5)	24 (3.6)	39 (6.8)	15 (2.2)	44 (6.5)	17 (2.5)
and others						
Income	*p* = 0.003^*^^a^		*p* = 0.508		*p* = 0.029^*^^a^	
Below 5,000	43 (6.3)	56 (8.3)	84 (12.4)	15 (2.2)	76 (11.2)	23 (3.4)
5,001–10,000	76 (11.2)	62 (9.1)	108 (15.9)	30 (4.4)	98 (14.5)	40 (5.9)
10,001–20,000	86 (12.7)	68 (10.0)	116 (17.1)	38 (5.6)	104 (15.3)	50 (7.4)
20,001–40,000	82 (12.1)	54 (8.0)	105 (15.5)	31 (4.6)	84 (12.4)	52 (7.7)
40,001–60,000	40 (5.9)	17 (2.5)	40 (5.9)	17 (2.5)	37 (5.5)	20 (2.9)
Above 60,000	57 (8.4)	37 (5.5)	78 (911.5)	16 (2.4)	61 (9.0)	33 (4.9)

* *Significant: P < 0.05*.

a *Chi-Square for trend*.

b *Pearson Chi-Square*.

*Participants 678, Chi-square test, was performed between trend with demographic characteristics*.

## Discussion

Worldwide, many countries have investigated several physiological and public health measures to mitigate the spread of COVID-19. The most frequently implemented action was face masks (9). Physiological preventive measures and public health strategies played a significant role in combatting infectious diseases, including the COVID-19 pandemic. These preventative measures are handwashing/sanitizing, social distance, avoiding public gatherings, and wearing a face mask (10). Understanding the predictors of increased adherence to these public health measures is essential for the COVID-19 and future pandemics. In this study, most participants had good knowledge about face masks and showed a positive attitude. The respondents believed that everyone could use the face mask. Saudi citizens have above-average knowledge and optimistic attitudes toward the use of face masks during the COVID-19 pandemic. The community is convinced about using face masks and believes that face masks play a predominant role in limiting the spread of the viral infection, SARS-CoV-2.

In this study, the use of face masks was significantly linked with age, gender, region, education, and employment. The young adults (16–34 years), male, higher education, and students showed better compliance than other groups. Similar findings have also been reported by Li et al. face masks use with social distancing could effectively minimize the COVID-19 risk in the community (11). Despite improper compliance, it is a fact that using any face mask decreases the disease exposure and the risk of infection (12). It has also been demonstrated that the community benefit of using a face mask depends on compliance and early use during the pandemic, together with hand hygiene (13).

A study on a Malaysian community has reported an overall knowledge rate of 80.5%, and most participants (83.1%) demonstrated positive attitudes toward preventing COVID-19 (11). In the present study, over 56.6% of the participants had a good knowledge of face masks, and more than half of the respondents, 78.3%, showed a positive attitude. The study observed a good practice score of 67.8% in the majority of the participants, with an average score. Most of the respondents, 70.2%, believed that everyone could use the face mask to minimize the spread of the disease. Sikakulya et al. (14) reported that about 60% of the participants had adequate knowledge on the use of face masks among the respondents; about 83.4% believed that a face mask could protect against COVID-19. The majority of respondents (95.2%) agreed that wearing face masks in public places was essential to protect themselves against COVID-19.

Alremeithi et al. (15) explored the population's knowledge, attitude, and practice toward COVID-19 in the United Arab Emirates. The authors reported that the knowledge was linked with higher education, and there was a positive association between the level of education and SARS-COV-2 infection knowledge. Larebo et al. (16) demonstrated that the student's overall knowledge was 29.2%, attitude 88.1%, and their practice was 89.5%. The students from the college natural and computational sciences have good knowledge independently associated with face mask utilization. While comparing with other studies, authors found that face masks usage knowledge was low, but the attitudes and practices were high.

Sugimura et al. (17) conducted a study on the relative risk of COVID-19 for mask users vs. non-mask users. In this study, about 800 participants were included; 53% wore masks. The non-mask users were infected at 16%, while mask users were infected at 7.1%. People using the face mask are 60% less likely to be infected with COVID-19 than non-mask wearers (17). In another study, findings also suggest that face mask use in public could help in mitigating the spread of COVID-19 (18). Face masks are highly essential to minimize the spread of infectious diseases such as the COVID-19 pandemic. The primary mechanism behind the use of face masks and their role in reducing the spread of SARS-CoV-2 disease is that face masks can reduce the entry of small tiny particles and air pollutants. The cluster of reports indicates that respiratory transmission is the primary source of transmission risk of SARS-CoV-2 (3). However, gastrointestinal (19, 20) and ocular (21) sources also transmit the diseases. Once these contaminated droplets and particles are on any surface, the air pollutants, mainly the particulate matter PM2.5, can easily carry and transport from region to region and easily contaminate an individual. A series of reports are available on the air pollutants and their role in spreading SARS-CoV-2 cases by Meo et al. (22–24). The face masks could minimize the entry of contaminants into the respiratory and oral cavity, minimizing the spread of the disease. We believe using face masks to reduce the spread of the diseases is essential for both the public and policymakers to combat against COVID-19 pandemic.

## Strengths and Limitations

This study's strength is that the findings are based on an appropriate sample size during the COVID-19 pandemic in Saudi Arabia. Second: the questionnaire survey was offered in Arabic and English languages, able to reach the large segments of the society containing both male and females population of the country. This study has some limitations. The social media group users are mainly the young and middle-aged generation, so the old age group participants may not represent this study appropriately. The second limitation is that the elderly and daily basis laborers could have been underestimated in our sample. The third limitation is that self-reported questions may not assess the findings appropriately; hence, further studies can be conducted to validate the results further and clarify the casual relationship to improve the population knowledge attitude and practice regarding the implementation of evidence-based effective physiological and public health policies and procedures as face masks.

## Conclusions

The results revealed good compliance of people in wearing face masks in public and workplaces in Saudi Arabia. The majority of the participants had good knowledge about face masks, and about two-thirds of respondents showed a positive attitude. Most of the respondents believed that everyone could use the face mask; however, a limited number reported that they would not wear it if the government did not recommend wearing it in public places during the COVID-19 pandemic. Saudi citizens have above-average knowledge and optimistic attitudes toward the use of face masks during the COVID-19 pandemic. The community is convinced about the use of face masks and believes that face masks play a significant role in limiting the spread of the SARS-CoV-2 infection.

## Data Availability Statement

The raw data supporting the conclusions of this article will be made available by the authors, without undue reservation.

## Ethics Statement

The studies involving human participants were reviewed and approved by Institutional Review Board approved this study, College of Medicine Research Centre, King Saud University, Riyadh, Saudi Arabia (Ref. E-21-6125). Written informed consent to participate in this study was provided by the participants' legal guardian/next of kin.

## Author Contributions

SM: project supervision, writing-review, and editing. SA, GA, FB, RAA, and RMA: data collection, checking, entry, and analysis. All authors contributed to the article and approved the submitted version.

## Conflict of Interest

The authors declare that the research was conducted in the absence of any commercial or financial relationships that could be construed as a potential conflict of interest.

## Publisher's Note

All claims expressed in this article are solely those of the authors and do not necessarily represent those of their affiliated organizations, or those of the publisher, the editors and the reviewers. Any product that may be evaluated in this article, or claim that may be made by its manufacturer, is not guaranteed or endorsed by the publisher.
